# The use of personal health information outside the circle of care: consent preferences of patients from an academic health care institution

**DOI:** 10.1186/s12910-021-00598-3

**Published:** 2021-03-24

**Authors:** Sarah Tosoni, Indu Voruganti, Katherine Lajkosz, Flavio Habal, Patricia Murphy, Rebecca K. S. Wong, Donald Willison, Carl Virtanen, Ann Heesters, Fei-Fei Liu

**Affiliations:** 1 Radiation Medicine Program, Princess Margaret Cancer Centre, Toronto, ON Canada; 2 Department of Radiation Oncology, University of Toronto, Toronto, ON Canada; 3 Department of Biostatistics, Princess Margaret Cancer Centre, Toronto, ON Canada; 4 Department of Medicine, University Health Network, Toronto, ON Canada; 5 Department of Anaesthesia, University Health Network, Toronto, ON Canada; 6 Institute of Health Policy, Management and Evaluation, University of Toronto, Toronto, ON Canada; 7 University Health Network Digital, Toronto, ON Canada; 8 Department of Bioethics, University Health Network, Toronto, ON Canada; 9 Joint Centre for Bioethics, University of Toronto, Toronto, ON Canada; 10 Princess Margaret Cancer Centre, Department of Radiation Oncology, 700 University Avenue, Toronto, ON M5G 2M9 Canada

**Keywords:** Patient consent preferences, Data sharing, Consent policies

## Abstract

**Background:**

Immense volumes of personal health information (PHI) are required to realize the anticipated benefits of artificial intelligence in clinical medicine. To maintain public trust in medical research, consent policies must evolve to reflect contemporary patient preferences.

**Methods:**

Patients were invited to complete a 27-item survey focusing on: (a) broad versus specific consent; (b) opt-in versus opt-out approaches; (c) comfort level sharing with different recipients; (d) attitudes towards commercialization; and (e) options to track PHI use and study results.

**Results:**

222 participants were included in the analysis; 83% were comfortable sharing PHI with researchers at their own hospital, although younger patients (≤ 49 years) were more uncomfortable than older patients (50 + years; 13% versus 2% uncomfortable, *p* < 0.05). While 56% of patients preferred broad consent, 38% preferred specific consent; 6% preferred not sharing at all. The majority of patients (63%) preferred to be asked for permission before entry into a contact pool. Again, this trend was more pronounced for younger patients (≤ 49 years: 76%). Approximately half of patients were uncomfortable sharing PHI with commercial enterprises (51% uncomfortable, 27% comfortable, 22% neutral). Most patients preferred to track PHI usage (61%), with the highest proportion once again reported by the youngest patients (≤ 49 years: 71%). A majority of patients also wished to be notified regarding study results (70%).

**Conclusions:**

While most patients were willing to share their PHI with researchers within their own institution, many preferred a transparent and reciprocal consent process. These data also suggest a generational shift, wherein younger patients preferred more specific consent options. Modernizing consent policies to reflect increased autonomy is crucial in fostering sustained public engagement with medical research.

**Supplementary Information:**

The online version contains supplementary material available at 10.1186/s12910-021-00598-3.

## Background

Rapid advancements in machine learning and artificial intelligence (AI) have generated a pressing need for massive volumes of personal health information (PHI). In an effort to capitalize on these technologies for medical discovery and innovation, some health organizations have encountered high-profile public scrutiny and/or legal action for sharing even de-identified health information outside the circle of care, with patients contesting claims that they actually provided *informed* consent, and questioning whether *de-identification* is truly possible [1, 2]. Furthermore, patients are becoming aware of potentially controversial PHI uses (e.g. for gene editing, and some AI applications), and some are noting the increasing monetary value of PHI (e.g. to commercial companies) [3–5].

Indeed, the use of PHI for research *without explicit consent* is occurring [3, 6], leading some health law experts to raise the concern that consent issues are poised to become a major social controversy [4, 7]. While some provisions in law permit the use of de-identified PHI for research without explicit consent, previous studies report that many patients do not distinguish between identifiable versus de-identified or anonymized data, and that they wish to be asked for consent even when PHI is de-identified [8, 9]. Proper informed consent is the foundation upon which trusting relationships are built; a well-recognized principle in health law, and prominent feature of international codes of ethics including the Declaration of Helsinki and the Nuremburg Code [10–13]. Beyond institutional privacy obligations, the duty of physicians to protect the privacy of their patients is sacrosanct, enshrined in normative documents including the Hippocratic Oath and professional codes of ethics [14]. While advocates for non-consensual access to PHI for research do exist, many scholars have been unequivocal that further public engagement is crucial to ensure the use of PHI is ethically defensible and patient-centered [4, 9, 10, 15–17].

Patient preferences for consent have been empirically studied for upwards of two decades, illuminating a diverse array of needs and views. In a large-scale European study, 61% of participants were concerned over third party access to PHI; participants were strongly averse to sharing with health insurance and private pharmaceutical companies [18]. In studies by Willison and by Joly, Canadian patients also expressed significant concern over commercial uses of PHI [9, 15, 19]; with 57% indicating that de-identified PHI should not be used at all for marketing/commercial purposes [15]. Multiple studies also report that substantial majorities of patients wish to be consulted before their de-identified PHI is used for research purposes [16, 20, 21], even after being fully informed of the potential drawbacks such as less research being conducted [14]. Furthermore, many patients desired more specific options beyond one-time broad consent [16, 22–24]; in a comprehensive systematic review “the most notable finding is that many people do not favor broad consent for either research itself or for research and subsequent wide data sharing [24].” Taken together, these reports illuminate a consistent trend that if PHI is to be used outside one’s own care, the opportunity for information control is considered an important element of respect for persons [8, 15].

While these studies have amplified patient voices within medical literature, policy changes have lagged, resulting in an unfortunate chasm between knowledge and practice. Indeed, previous studies have noted that healthcare organizations have a pervasive tendency to disregard mounting evidence that patients desire more information and control [3, 25, 26]. Therefore, the purpose of the current study was to acquire an in-depth understanding of the contemporary and specific consent needs of cancer patients at a large academic hospital to inform its own institutional consent policies.

## Methods

### Participants and procedure

A clinical research coordinator (CRC) invited patients in the waiting rooms of five radiation oncology clinics (breast, prostate, lung, thyroid, palliative) to complete a 10-min iPad survey (“Appendix 1”) from January to December, 2019. Full Research Ethics Board (REB) approval was granted from the University Health Network (UHN). The only inclusion criterion was being a patient at the cancer centre, and consent was provided verbally. Completed surveys were stored separately, not linked to patients’ names or medical records.

### Survey

An original 27-item survey was developed specifically for this study, and comprised of four sections of multiple choice and 5-point Likert-scale questions. The survey was designed in consultation with a diverse array of experts from medicine, digital technology, bioethics, and public health policy. It was reviewed in-person with three patient partners to ensure readability and clarity. The welcome message was vital to protect the integrity of responses from a health literacy perspective (“Appendix 1”); we could not assume that all patients would be aware that their PHI holds value outside their own care and is sought-after by external sources. Five sections followed; *Section 1: Asking for your Consent to Share; 2: Recipients; 3: Commercialization; 4: Tracking Sharing & Results; and 5: Background Questions*.

### Sample size justification

As a proof-of-concept survey study, sample size was chosen by convenience, hence statistical power calculation was not necessary. Our targeted sample size of 50 participants within each disease site would provide a confidence interval range of the preference rate (95% CI: 0.36–0.64) as 0.28 with high accuracy.

### Statistical analysis

Patient demographics were summarized using descriptive statistics. Distribution of responses to study questions were summarized using frequencies and percentages. Missing responses were not included in the denominator when calculating percentages. Results were also stratified by demographics to assess differences between groups, and the Chi-square test was utilized post-hoc to identify statistically significant differences between groups. Clinics were collapsed into palliative versus curative to identify potential differences based on prognosis. Analyses were conducted using SAS version 9.4 (Cary, NC). For the Chi-square analyses, the 5-point scales for questions 7 to 17 were collapsed to 3-point scales, due to small cell size in some sub-groups.

## Results

A total of 272 patients agreed to participate, with 222 (82%) completing the survey. The 18% who did not complete it were interrupted due to the timing of their appointment, or they expressed difficulty in understanding the questions. Approximately 70% of those approached agreed to participate. Our demographic reporting (Table 1) was limited to age, gender, treatment stage, and clinic due to a substantial proportion of patients being reluctant to share income and education information, and patient-reported ambiguity over the classification of ethnicity (e.g. many fit within multiple categories).

**Table 1:** Patient demographics of the 222 study participants

Demographic	N (%)
Age	
≤ 49	38 (17)
50–74	132 (59)
≥ 75	48 (21)
Rather not say	4 (2)
Gender	
Male	112 (50)
Female	108 (49)
Rather not say	2 (1)
Treatment stage	
Pre-treatment	22 (10)
Treatment	87 (40)
Follow-up	113 (50)
Clinic	
Breast	49 (22)
Lung	45 (20)
Prostate	49 (22)
Thyroid	48 (22)
Palliative	25 (11)
Other	6 (3)

Survey responses are summarized in Table 2, categorized by age, and palliative versus curative clinic. Additional file 1: Supplementary Table 1 presents the results (Q7-17) collapsed into 3-levels (i.e. one level combining very comfortable and comfortable responses, one level for neutral, and one level combining uncomfortable and very uncomfortable) with the same categories. Additional file 1: Supplementary Table 2 presents the responses categorized by individual clinic, treatment stage, and gender.

**Table 2:** Response results to survey questions

Question		Response	Overall N = 222	Age ≤ 49 N = 38	Age 50–74 N = 132	Age ≥ 75 N = 48	Curative Clinic N = 197	Palliative Clinic N = 25
Q1	Your health information is divided into several different sections (e.g. diagnoses test results and images such as x-rays or scans). Would you like to:	Share all information	177 (81)	26 (68)	111 (86)	38 (79)	154 (79)	23 (92)
		Share no information	12 (5)	4 (11)	4 (3)	3 (6)	12 (6)	0 (0)
		Share specific information	30 (14)	8 (21)	14 (11)	7 (15)	28 (14)	2 (8)
		No response	3	0	3	0	3	0
Q2	Your biological samples are classified into several different types (e.g. blood urine tissues). Would you like to:	Share all information	173 (79)	25 (66)	107 (83)	39 (81)	151 (78)	22 (88)
		Share no information	18 (8)	5 (13)	9 (7)	3 (6)	18 (9)	0 (0)
		Share specific information	28 (13)	8 (21)	13 (10)	6 (13)	25 (13)	3 (12)
		No response	3	0	3	0	3	0
Q3	There are many different areas of medical research (e.g. research on cancer diabetes reproductive disorders genetic disorders heart disease etc.). Would you like to:	Share all information	172 (79)	25 (66)	105 (81)	40 (85)	152 (78)	20 (83)
		Share no information	9 (4)	2 (5)	5 (4)	1 (2)	9 (5)	0 (0)
		Share specific information	37 (17)	11 (29)	19 (15)	6 (13)	33 (17)	4 (17)
		No response	4	0	3	1	3	1
Q4	When asked for consent to share your information or samples would you like to have an option to think about the decision and be asked again later?	Yes	107 (49)	22 (58)	65 (50)	18 (38)	97 (50)	10 (42)
		No	111 (51)	16 (42)	64 (50)	29 (62)	97 (50)	14 (58)
		No response	4	0 (0)	3	1	3	1
Q5	Your health information and samples are often requested for future studies. Would you like to:	Broad consent	119 (56)	16 (42)	69 (55)	33 (72)	103 (54)	16 (67)
		Study-specific consent	80 (38)	19 (50)	49 (39)	11 (24)	74 (39)	6 (25)
		Would not share	14 (6)	3 (8)	8 (6)	2 (4)	12 (6)	2 (8)
		No response	9	0	6	2	8	1
Q6	A CONTACT POOL may be created with patient names phone numbers and key pieces of health information. UHN Researchers with ethical approval for their studies could search this pool to find participants. Would you prefer to be:	Asked for permission	134 (63)	29 (76)	74 (59)	28 (61)	127 (66)	7 (30)
		Automatically entered	80 (37)	9 (24)	52 (41)	18 (39)	64 (34)	16 (70)
		No response	8	0	6	2	6	2
Q7	How comfortable are you with providing consent for your information or samples to be shared with Researchers within UHN?	Very Comfortable	121 (55)	17 (45)	76 (58)	26 (55)	108 (55)	13 (52)
		Comfortable	62 (28)	10 (26)	40 (30)	11 (23)	51 (26)	11 (44)
		Neutral	30 (14)	6 (16)	14 (11)	10 (21)	29 (15)	1 (4)
		Uncomfortable	5 (2)	3 (8)	2 (2)	0 (0)	5 (3)	0 (0)
		Very Uncomfortable	3 (1)	2 (5)	0 (0)	0 (0)	3 (2)	0 (0)
		No response	1	0	0	1	1	0
Q8	How comfortable are you with providing consent for your information or samples to be shared with Researchers at other hospital-based research institutes?	Very Comfortable	85 (38)	12 (32)	52 (39)	19 (40)	76 (39)	9 (36)
		Comfortable	68 (31)	9 (24)	45 (34)	14 (29)	55 (28)	13 (52)
		Neutral	44 (20)	11 (29)	24 (18)	9 (19)	42 (21)	2 (8)
		Uncomfortable	17 (8)	3 (8)	10 (8)	3 (6)	16 (8)	1 (4)
		Very Uncomfortable	8 (4)	3 (8)	1 (1)	3 (6)	8 (4)	0 (0)
Q9	How comfortable are you with providing consent for your information or samples to be shared with Researchers at universities?	Very Comfortable	71 (33)	11 (29)	45 (35)	13 (28)	65 (33)	6 (27)
		Comfortable	82 (38)	14 (37)	50 (39)	18 (38)	67 (34)	15 (68)
		Neutral	39 (18)	9 (24)	21 (16)	8 (17)	39 (20)	0 (0)
		Uncomfortable	18 (8)	1 (3)	11 (9)	6 (13)	18 (9)	0 (0)
		Very Uncomfortable	8 (4)	3 (8)	2 (2)	2 (4)	7 (4)	1 (5)
		No response	4	0	3	1	1	3
Q10	How comfortable are you with providing consent for your information or samples to be shared with For-profit businesses (e.g. drug or insurance companies such as Pfizer)?	Very Comfortable	35 (16)	6 (16)	20 (15)	8 (17)	32 (16)	3 (13)
		Comfortable	24 (11)	3 (8)	15 (12)	5 (11)	19 (10)	5 (22)
		Neutral	49 (22)	12 (32)	25 (19)	12 (26)	42 (21)	7 (30)
		Uncomfortable	51 (23)	7 (18)	33 (25)	11 (23)	44 (22)	7 (30)
		Very Uncomfortable	60 (27)	10 (26)	37 (28)	11 (23)	59 (30)	1 (4)
		No response	3	0	2	1	1	2
Q11	How comfortable are you with providing consent for your information or samples to be shared with Not-for-profit businesses (e.g. Heart and Stroke Foundation of Canada)?	Very Comfortable	64 (29)	9 (24)	41 (32)	12 (26)	60 (31)	4 (16)
		Comfortable	61 (28)	6 (16)	36 (28)	19 (40)	46 (24)	15 (60)
		Neutral	50 (23)	12 (32)	25 (19)	12 (26)	48 (25)	2 (8)
		Uncomfortable	31 (14)	9 (24)	20 (15)	2 (4)	28 (14)	3 (12)
		Very Uncomfortable	13 (6)	2 (5)	8 (6)	2 (4)	12 (6)	1 (4)
		No response	3	0	2	1	3	0
Q12	How comfortable are you with providing consent for your information or samples to be shared Provincially (i.e. within Ontario)?	Very Comfortable	52 (24)	7 (18)	33 (25)	10 (21)	47 (24)	5 (20)
		Comfortable	65 (30)	8 (21)	39 (30)	18 (38)	50 (26)	15 (60)
		Neutral	58 (26)	14 (37)	34 (26)	10 (21)	56 (29)	2 (8)
		Uncomfortable	28 (13)	6 (16)	16 (12)	6 (13)	26 (13)	2 (8)
		Very Uncomfortable	16 (7)	3 (8)	8 (6)	3 (6)	15 (8)	1 (4)
		No response	3	0	2	1	3	0
Q13	How comfortable are you with providing consent for your information or samples to be shared Nationally (i.e. within Canada)?	Very Comfortable	52 (24)	7 (18)	35 (27)	9 (19)	47 (24)	5 (20)
		Comfortable	64 (29)	9 (24)	38 (29)	16 (34)	50 (26)	14 (56)
		Neutral	57 (26)	13 (34)	33 (25)	11 (23)	54 (28)	3 (12)
		Uncomfortable	29 (13)	5 (13)	16 (12)	7 (15)	27 (14)	2 (8)
		Very Uncomfortable	17 (8)	4 (11)	8 (6)	4 (9)	16 (8)	1 (4)
		No response	3	0	2	1	3	0
Q14	How comfortable are you with providing consent for your information or samples to be shared Internationally (i.e. around the world)?	Very Comfortable	44 (20)	7 (18)	28 (22)	8 (17)	40 (21)	4 (16)
		Comfortable	41 (19)	7 (18)	26 (20)	8 (17)	30 (15)	11 (44)
		Neutral	53 (24)	11 (29)	29 (22)	13 (28)	49 (25)	4 (16)
		Uncomfortable	46 (21)	6 (16)	29 (22)	10 (21)	42 (22)	4 (16)
		Very Uncomfortable	35 (16)	7 (18)	18 (14)	8 (17)	33 (17)	2 (8)
		No response	3	0	2	1	3	0
Q15	Sometimes for-profit companies develop partnerships with UHN and we work together on medical research projects. How comfortable are you consenting to share your information or samples for these projects?	Very Comfortable	38 (17)	6 (16)	25 (19)	6 (13)	35 (18)	3 (12)
		Comfortable	73 (33)	11 (29)	43 (33)	18 (38)	57 (29)	16 (64)
		Neutral	48 (22)	9 (24)	28 (21)	11 (23)	45 (23)	3 (12)
		Uncomfortable	33 (15)	6 (16)	18 (14)	8 (17)	30 (15)	3 (12)
		Very Uncomfortable	30 (14)	6 (16)	18 (14)	5 (10)	30 (15)	0 (0)
Q16	Sometimes for-profit companies ask UHN for health information or samples. How comfortable are you consenting to share your information or samples with these companies if UHN is not directly involved in their work?	Very Comfortable	25 (12)	2 (6)	17 (13)	6 (13)	22 (12)	3 (12)
		Comfortable	43 (20)	6 (17)	29 (22)	7 (15)	34 (18)	9 (36)
		Neutral	37 (17)	7 (20)	17 (13)	13 (28)	35 (18)	2 (8)
		Uncomfortable	53 (25)	10 (29)	34 (26)	9 (20)	43 (23)	10 (40)
		Very Uncomfortable	57 (27)	10 (29)	34 (26)	11 (24)	56 (29)	1 (4)
		No response	7	3	1	2	7	0
Q17	Sometimes medical research using health information or samples at UHN leads to discoveries that are commercialized and sold for-profit in the future. How do you feel about consenting to share your information or samples being involved in this?	Very Comfortable	29 (13)	4 (11)	19 (15)	6 (13)	25 (13)	4 (17)
		Comfortable	52 (24)	6 (16)	32 (24)	12 (26)	42 (21)	10 (42)
		Neutral	51 (23)	11 (29)	33 (25)	7 (15)	48 (24)	3 (13)
		Uncomfortable	47 (21)	8 (21)	25 (19)	14 (30)	41 (21)	6 (25)
		Very Uncomfortable	41 (19)	9 (24)	22 (17)	8 (17)	40 (20)	1 (4)
		No response	2	0	1	1	1	1
Q18	Would you like to be able to track who is using your information or samples and what they are using it for?	Yes	134 (61)	27 (71)	83 (63)	22 (47)	124 (63)	10 (40)
		No	77 (35)	7 (18)	45 (34)	24 (51)	63 (32)	14 (56)
		Not applicable. I would not share at all	10 (5)	4 (11)	4 (3)	1 (2)	9 (5)	1 (4)
		No response	1	0	0	1	1	0
Q19	Would you like to be notified with the results of studies that have used your information or samples?	Yes	155 (70)	29 (76)	96 (73)	28 (58)	140 (71)	15 (60)
		No	57 (26)	5 (13)	32 (24)	19 (40)	48 (24)	9 (36)
		Not applicable. I would not share at all	10 (5)	4 (11)	4 (3)	1 (2)	9 (5)	1 (4)
Q20	If you do want to be notified of study results how would you like to be notified?	Online via an electronic patient portal	76 (35)	15 (39)	51 (39)	8 (17)	66 (34)	10 (40)
		Online via email	50 (23)	14 (37)	29 (22)	7 (15)	47 (24)	3 (12)
		Standard mail	43 (20)	3 (8)	25 (19)	15 (31)	39 (20)	4 (16)
		I do NOT want to be notified of study results	40 (18)	4 (11)	20 (15)	15 (31)	34 (17)	6 (24)
		Not applicable. I would not share at all	11 (5)	2 (5)	5 (4)	3 (6)	9 (5)	2 (8)
		No response	2	0	2	0	2	0

**Fig. 1:**
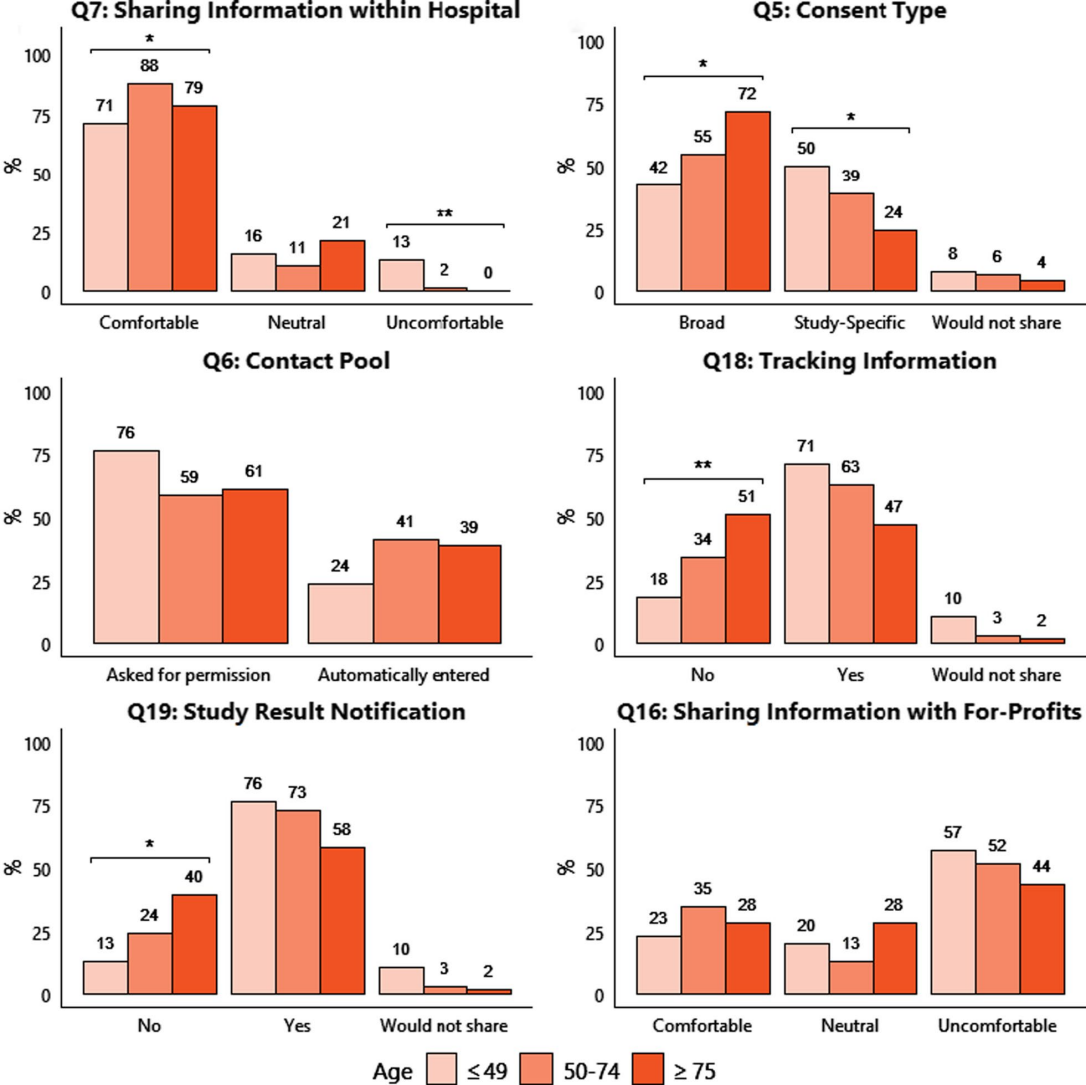
Results stratified by age for select questions. Annotated bar chart showing the percentage of participants with each response for the listed question, categorized by age group (≤ 49 years, 50–74 years, ≥ 75 years). Missing responses are not included. Q = Question. *p < 0.05; **p < 0.01

Overall, the majority of patients reported being comfortable with sharing their health information with their own institutional (UHN) researchers (Table 2; Q7—83%, *p* < 0.001). Palliative patients were slightly more comfortable compared to curative patients (96% vs. 81%). Younger patients (≤ 49 years) were more uncomfortable than older patients (50 + years) with sharing even within their own hospital (13% vs. 2%, *p* < 0.05; Fig. 1a). More patients preferred a broad consent strategy over a study-specific strategy (Table 2; Q5—56% vs. 38%), although 6% would not share at all. This trend was reported by patients aged 50–74 years (55% broad vs. 39% study-specific consent), and patients ≥ 75 years (72% broad vs. 24% study-specific consent), but reversed in younger patients aged ≤ 49 years (42% broad vs. 50% study-specific consent, *p* < 0.05; Fig. 1b).

When categorized by age, several additional trends were observed (Fig. 1). The majority of patients (63%, *p* < 0.001) wished to be asked for permission before being entered into a contact pool (Q6). This was particularly notable for younger patients aged ≤ 49 years at 76% (Fig. 1c). Additionally, most patients preferred the option to track PHI usage (Q18—61%, *p* < 0.05), with the highest proportion again reported in the youngest patient group (≤ 49 years: 71%; 50–75 years: 63%; 75 + years: 47%, *p* < 0.01; Fig. 1d). The most preferred method of tracking (Q20) was via an online electronic patient portal (35%), followed by email (23%), then standard mail (20%). A majority of patients preferred to receive results of studies that have used their information or samples (Q19—70%, *p* < 0.001). Once again, the highest proportion was observed in the youngest patients (≤ 49 years: 76%; 50–74 years: 73%; 75 + years: 58%, *p* < 0.05; Fig. 1e). This preference remained stable regardless of palliative (60%) or curative (71%) status. Interestingly, patients in the follow-up clinic were significantly more likely to wish to track their PHI use (Q18—72%; *p* < 0.01, and receive study results (Q19—81%; *p* < 0.01) as compared to those in pre-treatment or treatment groups (Additional file 1: Table 2). In terms of selectivity, 14% of patients preferred the option to select parts of their health information to share (Q1), 13% wished to select specific components of their biological samples to share (Q2), and 17% desired the option to select specific areas of research to share (Q3) (all Table 2). Females were significantly more likely to select components of health information to share than males (Q1—20% vs. 7%; *p* < 0.01; Additional file 1: Table 2).

**Fig. 2:**
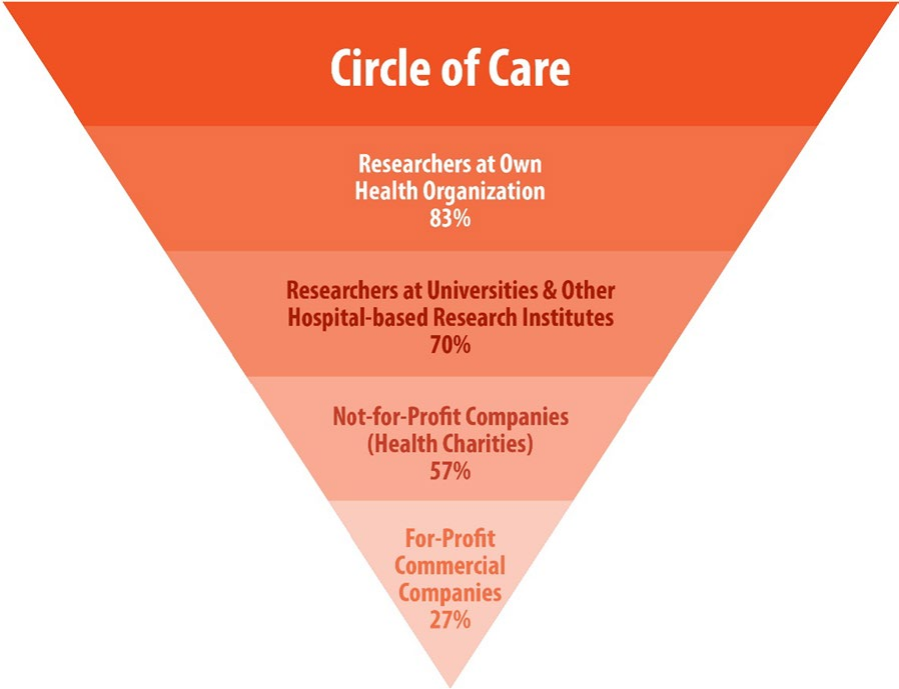
Percentage of patients reporting comfort sharing PHI as a function of recipient. Inverted pyramid chart showing the progressive decline in percentage of participants reporting comfort sharing with recipients outside the circle of care from within one’s own health organization (83%) to for-profit commercial companies (27%)

Intended recipient of PHI was a very important consideration. Few patients overall were comfortable sharing with commercial companies (Q10—27%); with 51% reporting being uncomfortable, and 22% offering neutral responses. This is clearly illustrated in Fig. 2, whereby the percentage of patients reporting comfort as a function of recipients varied significantly. Comfort was highest with respect to sharing with researchers at the respondents’ own health organization (83%), but dropped to 70% for academic researchers, 57% for non-profit companies, and plummeted to 27% with for-profit commercial companies. When the host institution had a collaborative partnership with the companies, 50% of patients were comfortable, 22% neutral, and 28% were uncomfortable sharing (Q15). Without direct institutional involvement however, only 32% were comfortable, 17% neutral, and 51% were uncomfortable in sharing with commercial companies (Q16). Once again, younger patients (≤ 49 years) were more likely to report discomfort in sharing PHI with commercial companies without host institution involvement compared to the oldest group (75 + years; 57% vs. 43%) (Fig. 1f). Significant differences were reported for Q16 by gender and treatment stage (Additional file 1: Table 2). Females were more likely to be uncomfortable than comfortable (57% vs. 44%, *p* < 0.05), whereas males were more likely to be comfortable than uncomfortable (40% vs. 23%, *p* < 0.01). Finally, significantly more patients in the follow-up stage were uncomfortable (60%) when compared to pre-treatment (50%) or active treatment (40%; *p* < 0.05) cohorts.

## Discussion

The vast majority (83%) of participants were comfortable sharing PHI with researchers at their own institution, while most wished to be asked first and apprised of its potential uses. This observation is reassuring, and reflects a generally positive attitude towards locally conducted research. The fact however, that a minority (17%) did not report comfort sharing PHI with even host institutional researchers is noteworthy, particularly since these participants had already *agreed to complete a research survey*. Though willingness to complete a research survey does not necessarily correlate with willingness to share PHI, it seems reasonable to suggest that those already participating in research may hold more favorable attitudes to sharing their PHI with researchers. Thus, reality may reflect a higher proportion of the patient population that is not comfortable sharing PHI even with local researchers. This underscores the importance of addressing this challenge in organizational policy, applicable to even low-risk retrospective studies.

One of our key observations relates to the effect of age on PHI sharing. Not only were younger patients more likely to report discomfort sharing with host institutional researchers and commercial companies without host institutional involvement, they were also more likely to prefer: (a) study-specific consent; (b) to be asked for permission before contact pool entry; (c) the ability to track PHI use; and (d) to receive study results. This is compelling evidence of a generational shift whereby younger patients raised in cultures of increasingly immersive technology (particularly more so than those 75 + years of age) are more hesitant to share, and desire more control over the use of their information. Our findings are consistent with previous observations that younger people prefer more control with respect to research participation than older individuals [18, 21, 24]. Whether these preferences will evolve over time remains to be seen, but it is possible that being raised in a technological era with platforms such as Facebook™ and Google™ renders this generation less trusting of institutions. Indeed, trust in institutions is commonly lowest amongst younger populations [27, 28]. It may be likely that this desire will be sustained as these individuals age and become major users of health care. It would be prudent, therefore, for health care organizations to acknowledge this trend, and to adapt consent policies correspondingly.

In our study, significantly more women preferred specific consent options and held higher levels of discomfort sharing PHI than did men (Q1; Q16), a finding which has been noted previously [19, 24]. Acknowledgement by health organizations of these gender differences is a step toward greater respect and inclusion; reforming existing policies to be more equitable and forward thinking would constitute significant progress.

Given that substantial groups of patients prefer study-specific consent (38%), and would like the opportunity to consider their consent decisions and be asked again later (49%), further exploration of how practicably to provide these options is warranted. These findings are consistent with previously reported studies [22, 23, 29, 30] reflecting many patients’ general reluctance for a broad consent or no-consent model.

Debate has also been ongoing regarding opt-in versus opt-out approaches for research contact pools. Some major health organizations employ or advocate for an opt-out approach whereby patient names and select PHI (e.g. age, gender, diagnosis, clinics & programs accessed, etc.) are automatically entered into databases, and patients can contact the institution to opt-out [31]. While this streamlines the process for researchers, some have challenged the ethical defensibility and legality of sharing potentially sensitive PHI outside the circle of care without active consent [20]. The current data provide strong support for an opt-in strategy, wherein a majority of respondents (63%), and an even greater majority of younger respondents (76%) preferred to be asked for permission before entry (Q6). This corroborates previously reported observations of patients preferring opt-in [9, 15, 21, 32]. To return to our earlier observation, it is particularly notable that the majority of patients *who already agreed to participate in a research survey* still wished to be asked permission before contact pool entry. One could reasonably infer that an even higher percentage of those not partaking in research surveys would prefer to be asked for permission. The success of medical research hinges on public trust in health institutions. Our findings lead us to assert that the precarious assumption of consent under an opt-out system risks undermining patients’ trust in research hospitals—trust that has been tested in a number of high profile cases and is not easily regained once eroded [1, 2].

The considerable level of patient discomfort with PHI being shared with for-profit commercial companies warrants broad attention. More than half of our patients (51%) reported being uncomfortable sharing with industry recipients (Q10; Fig. 2). This aligns with previous investigations in the cancer patient population, whereby low levels of trust towards industry and insurance recipients have also been reported [23]. While it appears that direct collaborations between health organizations and industry may mitigate some level of discomfort, it does not diminish it completely (51% to 28%). These results corroborate a significant and growing body of evidence that suggests that patients desire more transparency and control over commercial PHI use [9, 11, 15, 18–20, 32–38]. We recommend that patients be provided with an active opt-in choice as to whether they wish to share PHI with commercial companies. While this proposition is likely to face strong resistance within some research and industry circles, we submit that the continuation of a practice known to cause significant discomfort in the majority of patients is a governance misstep by healthcare organizations which purport to be committed to patient-centered care and respectful of the principle of patient self-determination. At the very least, it seems uncontroversial to suggest that patients should be given the option to choose whether or not their PHI can be shared with industry when their own health organization is *not* an active research partner.

Our results also reflect a substantial preference to be informed of secondary PHI use (61%), and to receive results of studies using their PHI (70%). This indicates that patients truly take an interest in research uses of PHI, and in clinical research findings in general. These observations again align with existing evidence; in a 2019 broad systematic review, participants almost never expected financial compensation, but “they demanded information on the research results” [30]. Admittedly, respecting this preference will require investment in technological tracking systems, but dynamic digital platforms do exist and should be employed [39]. Although significant work is required upfront, these investments promise significant yields in the long term; patients previously reluctant to share may become more willing if more information is provided. At minimum, health organizations could ensure that results are accessible through a publically available database. It is notable that higher proportions of patients in follow-up stages of care prefer to track PHI use (72%) and to receive study results (81%). To our knowledge, this is a novel finding indicating that as patients approach the end of their care journey, the desire for information not only remains, but becomes more pronounced. This underscores the enduring nature of this preference, and further emphasizes the need for health care organizations to provide meaningful options. It is also important to highlight that the majority of patients with a palliative diagnosis (60%; n = 25) also desired the option to receive study results. The opportunity to learn how one’s own PHI has contributed to the advancement of medical science may hold value and meaning for patients, including those at the end of life.

Our findings should be interpreted within the context of several limitations. Our participants were limited to those with cancer in radiation oncology clinics at a large academic centre, within a publically funded healthcare system. As such, we cannot be certain that preferences can be generalized to more diverse populations. We also acknowledge that patients may not have had extensive background knowledge on the benefits and drawbacks of each consent strategy. Although we endeavored to provide as much information as possible, we were cognizant of patients’ limited time, and kept the survey to a palatable 10-min. Finally, as we have noted, our findings reflect only the preferences of patients who were *willing to participate in a survey*. While we have attempted to extrapolate the implications for patients not interested in research participation, we acknowledge the more speculative nature of these observations.

## Conclusions

Our results underscore the importance of patient-centered consent and data governance policies that accurately reflect the identified preferences of patients. We note a consistent desire for greater transparency and autonomy, and our results particularly reveal a generational shift wherein younger patients prefer more informed consent options. Multiple factors must be considered including the consent process itself, the technological infrastructure to operationalize, and the nature of the broader healthcare system. Amongst the most readily achievable changes, we strongly recommend: (a) opt-in approaches to seek permission for research contact; and (b) the provision of genuine opportunities for uncertain patients to consider their consent decisions and revisit them later. While the following introduce more complexity, we also recommend providing options to choose: (a) broad or study-specific consent; (b) the conditions under which PHI may be shared with commercial companies; (c) whether to track secondary PHI use; and (d) whether to receive results of studies using PHI. We would be remiss to allow these results to reverberate in an esoteric echo chamber; further work must uncover strategies to translate these findings into effective policy changes. While this seems like a vast undertaking, the very technological advancements that have prompted the need for massive volumes of PHI can no doubt be applied to the design and implementation of modern consent platforms that exemplify a respect for well-identified patient values and preferences.

### Supplementary Information

**Additional file 1**. **Supplementary Table 1**. Results of Q7-17 collapsed into 3 levels. **Supplementary Table 2**. Responses categorized by individual clinic, treatment stage, and gender.

## Data Availability

The datasets used and/or analyzed during the current study are available from the corresponding author on reasonable request.

## Appendix 1

### Survey

Hello and welcome,

You are being invited to participate in this survey because you are a patient at the Princess Margaret Cancer Centre within the University Health Network (UHN). It will only take about 10 min and your answers are anonymous (i.e., not linked to your name or medical record).

During your care at UHN, health information (e.g., your medical record with diagnoses, test results, and images such as x-rays), and biological samples (e.g., blood, tissues, or bodily fluids drawn for medical procedures) are collected and stored. The main purpose of collecting and storing your information and samples is for your own treatment and care.

Your health information and biological samples are also very valuable for other purposes. They can be used to help find new ways to detect, treat, prevent, and possibly cure many health-related problems such as cancer, diabetes, and heart disease. Researchers within UHN, and at other hospital-based research institutes, universities, and health-related companies request access to your information and samples for these reasons. The purpose of this survey is to help us understand your preferences on the way we ask for your consent to share.

When you consent to sharing your health information or samples, security measures are in place to protect your privacy including removing your name and anything that could directly identify you. There is still a small risk that donated information or samples could be traced back to you.

The choice to do this survey is completely up to you. Your decision will not affect your treatment or care in any way. During the completion of the survey, you are free to withdraw at any time and your answers will be deleted. There are no known risks or direct benefits to participating. If you consent to participating in this survey, please answer the questions on the following pages.

Thank you very much!

## Section 1: Asking for your Consent to Share

1. Your health information is divided into several different sections (e.g., diagnoses, test results, and images such as x-rays or scans). Would you like to:
  - Share ALL of your health information for research
  - Share NONE of your health information for research
  - CHOOSE SPECIFIC PARTS of your health information to share for research
2. Your biological samples are classified into several different types (e.g., blood, urine, tissues). Would you like to:
  - Share ALL your biological samples for research
  - Share NONE of your biological samples for research
  - CHOOSE SPECIFIC PARTS of your biological samples to share for research
3. There are many different areas of medical research (e.g., research on cancer, diabetes, reproductive disorders, genetic disorders, heart disease, etc.). Would you like to:
  - Share with ALL areas of research
  - Share with NO areas of research
  - CHOOSE SPECIFIC AREAS of research to share with
4. When asked for consent to share your information or samples, would you like to have an option to think about the decision and be asked again later?
  - Yes
  - No
5. Your health information and samples are often requested for future studies. Would you like to:
  - Be asked for consent ONCE for ALL FUTURE STUDIES. You are not given study details
  - Be contacted with details about each study and asked for consent EACH TIME
  - Not Applicable. I would NOT share at all
6. A CONTACT POOL may be created with patient names, phone numbers, and key pieces of health information (e.g., “breast cancer” or “depression” or “family support clinic”). UHN Researchers with ethical approval for their studies could search this pool to find participants. Would you prefer to be:
  - ASKED FOR YOUR PERMISSION and only entered in the pool if you say yes
  - AUTOMATICALLY ENTERED in the pool. You are given a form with a number to call if you’d like to be removed

## Section 2: Recipients

With security measures in place to protect your privacy including removing your name and anything that could directly identify you (though there is still a small risk that your information or samples could be traced back to you), please tell us how comfortable you feel sharing your information or samples with the following recipients.

How comfortable are you with providing consent for your information or samples to be shared with **Researchers within UHN?**

- Very Comfortable
- Comfortable
- Neutral
- Uncomfortable
- Very Uncomfortable

How comfortable are you with providing consent for your information or samples to be shared with **Researchers at other hospital-based research institutes**?

- Very Comfortable
- Comfortable
- Neutral
- Uncomfortable
- Very Uncomfortable

How comfortable are you with providing consent for your information or samples to be shared with **Researchers at universities**?

- Very Comfortable
- Comfortable
- Neutral
- Uncomfortable
- Very Uncomfortable

How comfortable are you with providing consent for your information or samples to be shared with **For-profit businesses (e.g., drug or insurance companies such as Pfizer)**?

- Very Comfortable
- Comfortable
- Neutral
- Uncomfortable
- Very Uncomfortable

How comfortable are you with providing consent for your information or samples to be shared with **Not-for-profit businesses (e.g., Heart and Stroke Foundation of Canada)**?

- Very Comfortable
- Comfortable
- Neutral
- Uncomfortable
- Very Uncomfortable

How comfortable are you with providing consent for your information or samples to be shared **Provincially (i.e., within Ontario)?**

- Very Comfortable
- Comfortable
- Neutral
- Uncomfortable
- Very Uncomfortable

How comfortable are you with providing consent for your information or samples to be shared **Nationally (i.e., within Canada)?**

- Very Comfortable
- Comfortable
- Neutral
- Uncomfortable
- Very Uncomfortable

How comfortable are you with providing consent for your information or samples to be shared **Internationally (i.e., around the world)?**

- Very Comfortable
- Comfortable
- Neutral
- Uncomfortable
- Very Uncomfortable

## Section 3: Commercialization

When medical discoveries are made such as new treatments or cures for diseases, commercialization (i.e., selling for money) helps the discoveries to reach and help a great number of people. It also means that researchers and commercial companies may benefit financially. Patients who contribute their health information or samples do not get any money from this commercialization. We would like to understand how you feel about consenting to your health information or samples being used for projects that involve commercialization.

1. Sometimes for-profit companies (e.g., drug or insurance companies) develop partnerships with UHN and we work together on medical research projects. How comfortable are you consenting to share your information or samples (with your name and direct identifiers removed) for these projects?
  - Very Comfortable
  - Comfortable
  - Neutral
  - Uncomfortable
  - Very Uncomfortable
2. Sometimes for-profit companies (e.g., drug or insurance companies) ask UHN for health information or samples. How comfortable are you consenting to share your information or samples (with your name and direct identifiers removed) with these companies if UHN is not directly involved in their work?
  - Very Comfortable
  - Comfortable
  - Neutral
  - Uncomfortable
  - Very Uncomfortable
3. Sometimes medical research using health information or samples at UHN leads to discoveries that are commercialized and sold for-profit in the future. How do you feel about consenting to share your information or samples (with your name and direct identifiers removed) being involved in this?
  - Very Comfortable
  - Comfortable
  - Neutral
  - Uncomfortable
4. Very Uncomfortable

## Section 4: Tracking sharing & results

1. Would you like to be able to track who is using your information or samples and what they are using it for?
  - Yes
  - No
  - Not Applicable. I would not share at all
2. Would you like to be notified with the results of studies that have used your information or samples?
  - Yes
  - No
  - Not Applicable. I would not share at all
3. If you do want to be notified of study results, **how** would you like to be notified?
  - Online via an electronic patient portal (i.e., a secure website that allows you to track your personal health record)
  - Online via email
  - Standard mail
  - I do NOT want to be notified of study results
  - Not Applicable. I would not share at all

## Section 5: Background questions

Please answer the following questions on your background. Thank you very much for your participation!

1. Which clinic are you receiving treatment in?
  - Breast
  - Prostate
  - Lung
  - Thyroid
  - Other
2. What stage of your journey are you currently at?
  - Pre-treatment
  - Treatment
  - Follow-up
3. Age
  - 18–34
  - 35–49
  - 50–74
  - 75 + 
  - Rather not say
4. Sex
  - Male
  - Female
  - Other
  - Rather not say
5. Ethnicity
  - Canadian
  - European Canadian
  - African Canadian
  - Asian Canadian
  - First Nations
  - Other
  - Rather not say
6. Highest level of education
  - High school or less
  - Some post-secondary training (i.e., trade, college, university)
  - Completed trade/college diploma
  - Completed university degree
  - Completed post-graduate university degree (e.g., MA, MSc, PhD)
  - Rather not say
7. Household income ($)
  -  < 40,000/year
  - 40,000–60,000/year
  - 60,000–80,000/year
  - 80,000–100,000/year
  -  > 100,000 + /year
  - Rather not say

## Abbreviations

AI: Artificial intelligence
PHI: Personal health information
CRC: Clinical research coordinator
REB: Research Ethics Board
UHN: University Health Network
