# Supplementary material for: The use of personal health information outside the circle of care: consent preferences of patients from an academic health care institution
Source: BMC Med Ethics. 2021 Mar 24;22:29. doi: 10.1186/s12910-021-00598-3 (PMC7992944; doi:10.1186/s12910-021-00598-3)
Supplement: Supplementary file 1 — Additional file 1. Supplementary Table 1. Results of Q7-17 collapsed into 3 levels. Supplementary Table 2. Responses categorized by individual clinic, treatment stage, and gender. [file 12910_2021_598_MOESM1_ESM.docx]

**Supplementary Table 1**

**Response Results to Survey Questions – Comfort-level Questions (Q7-17) Collapsed into Three Levels**

| **Question** | | **Response** | **Overall N = 222** | **Age ≤ 49 N = 38** | **Age 50-74 N = 132** | **Age ≥ 75 N = 48** | **Curative Clinic N = 197** | **Palliative Clinic N = 25** |
| --- | --- | --- | --- | --- | --- | --- | --- | --- |
| **Q7** | How comfortable are you with providing consent for your information or samples to be shared with Researchers within UHN? | Comfortable | 183 (83) | 27 (71) | 116 (88) | 37 (79) | 159 (81) | 24 (96) |
|  |  | Neutral | 30 (14) | 6 (16) | 14 (11) | 10 (21) | 29 (15) | 1 (4) |
|  |  | Uncomfortable | 8 (4) | 5 (13) | 2 (2) | 0 (0) | 8 (4) | 0 (0) |
|  |  | No response | 1 | 0 | 0 | 1 | 1 | 0 |
| **Q8** | How comfortable are you with providing consent for your information or samples to be shared with Researchers at other hospital-based research institutes? | Comfortable | 153 (69) | 21 (55) | 97 (73) | 33 (69) | 131 (66) | 22 (88) |
|  |  | Neutral | 44 (20) | 11 (29) | 24 (18) | 9 (19) | 42 (21) | 2 (8) |
|  |  | Uncomfortable | 25 (11) | 6 (16) | 11 (8) | 6 (13) | 24 (12) | 1 (4) |
| **Q9** | How comfortable are you with providing consent for your information or samples to be shared with Researchers at universities? | Comfortable | 153 (70) | 25 (66) | 95 (74) | 31 (66) | 132 (67) | 21 (95) |
|  |  | Neutral | 39 (18) | 9 (24) | 21 (16) | 8 (17) | 39 (20) | 0 (0) |
|  |  | Uncomfortable | 26 (12) | 4 (11) | 13 (10) | 8 (17) | 25 (13) | 1 (5) |
|  |  | No response | 4 | 0 | 3 | 1 | 1 | 3 |
| **Q10** | How comfortable are you with providing consent for your information or samples to be shared with For-profit businesses (e.g. drug or insurance companies such as Pfizer)? | Comfortable | 59 (27) | 9 (24) | 35 (27) | 13 (28) | 51 (26) | 8 (35) |
|  |  | Neutral | 49 (22) | 12 (32) | 25 (19) | 12 (26) | 42 (21) | 7 (30) |
|  |  | Uncomfortable | 111 (51) | 17 (45) | 70 (54) | 22 (47) | 103 (53) | 8 (35) |
|  |  | No response | 3 | 0 | 2 | 1 | 1 | 2 |
| **Q11** | How comfortable are you with providing consent for your information or samples to be shared with Not-for-profit businesses (e.g. Heart and Stroke Foundation of Canada)? | Comfortable | 125 (57) | 15 (39) | 77 (59) | 31 (66) | 106 (55) | 19 (76) |
|  |  | Neutral | 50 (23) | 12 (32) | 25 (19) | 12 (26) | 48 (25) | 2 (8) |
|  |  | Uncomfortable | 44 (20) | 11 (29) | 28 (22) | 4 (9) | 40 (21) | 4 (16) |
|  |  | No response | 3 | 0 | 2 | 1 | 3 | 0 |
| **Q12** | How comfortable are you with providing consent for your information or samples to be shared Provincially (i.e. within Ontario)? | Comfortable | 117 (53) | 15 (39) | 72 (55) | 28 (60) | 97 (50) | 20 (80) |
|  |  | Neutral | 58 (26) | 14 (37) | 34 (26) | 10 (21) | 56 (29) | 2 (8) |
|  |  | Uncomfortable | 44 (20) | 9 (24) | 24 (18) | 9 (19) | 41 (21) | 3 (12) |
|  |  | No response | 3 | 0 | 2 | 1 | 3 | 0 |
| **Q13** | How comfortable are you with providing consent for your information or samples to be shared Nationally (i.e. within Canada)? | Comfortable | 116 (53) | 16 (42) | 73 (56) | 25 (53) | 97 (50) | 19 (76) |
|  |  | Neutral | 57 (26) | 13 (34) | 33 (25) | 11 (23) | 54 (28) | 3 (12) |
|  |  | Uncomfortable | 46 (21) | 9 (24) | 24 (18) | 11 (23) | 43 (22) | 3 (12) |
|  |  | No response | 3 | 0 | 2 | 1 | 3 | 0 |
| **Q14** | How comfortable are you with providing consent for your information or samples to be shared Internationally (i.e. around the world)? | Comfortable | 85 (39) | 14 (37) | 54 (42) | 16 (34) | 70 (36) | 15 (60) |
|  |  | Neutral | 53 (24) | 11 (29) | 29 (22) | 13 (28) | 49 (25) | 4 (16) |
|  |  | Uncomfortable | 81 (37) | 13 (34) | 47 (36) | 18 (38) | 75 (39) | 6 (24) |
|  |  | No response | 3 | 0 | 2 | 1 | 3 | 0 |
| **Q15** | Sometimes for-profit companies develop partnerships with UHN and we work together on medical research projects. How comfortable are you consenting to share your information or samples for these projects? | Comfortable | 111 (50) | 17 (45) | 68 (52) | 24 (50) | 92 (47) | 19 (76) |
|  |  | Neutral | 48 (22) | 9 (24) | 28 (21) | 11 (23) | 45 (23) | 3 (12) |
|  |  | Uncomfortable | 63 (28) | 12 (32) | 36 (27) | 13 (27) | 60 (30) | 3 (12) |
| **Q16** | Sometimes for-profit companies ask UHN for health information or samples. How comfortable are you consenting to share your information or samples with these companies if UHN is not directly involved in their work? | Comfortable | 68 (32) | 8 (23) | 46 (35) | 13 (28) | 56 (29) | 12 (48) |
|  |  | Neutral | 37 (17) | 7 (20) | 17 (13) | 13 (28) | 35 (18) | 2 (8) |
|  |  | Uncomfortable | 110 (51) | 20 (57) | 68 (52) | 20 (43) | 99 (52) | 11 (44) |
|  |  | No response | 7 | 3 | 1 | 2 | 7 | 0 |
| **Q17** | Sometimes medical research using health information or samples at UHN leads to discoveries that are commercialized and sold for-profit in the future. How do you feel about consenting to share your information or samples being involved in this? | Comfortable | 81 (37) | 10 (26) | 51 (39) | 18 (38) | 67 (34) | 14 (58) |
|  |  | Neutral | 51 (23) | 11 (29) | 33 (25) | 7 (15) | 48 (24) | 3 (13) |
|  |  | Uncomfortable | 88 (40) | 17 (45) | 47 (36) | 22 (47) | 81 (41) | 7 (29) |
|  |  | No response | 2 | 0 | 1 | 1 | 1 | 1 |

**Supplementary Table 2**

**Response Results to Survey Questions – Stratified by Clinic, Treatment Stage, Sex**

| **Question** | | **Response** | **Clinic** | | | | | | **Treatment Stage** | | | **Sex** | |
| --- | --- | --- | --- | --- | --- | --- | --- | --- | --- | --- | --- | --- | --- |
|  |  |  | **Breast N = 49** | **Prostate N = 45** | **Lung N = 49** | **Thyroid N = 48** | **Other N = 6** | **Palliative N = 25** | **Pre-Treatment N = 22** | **Treatment N = 87** | **Follow Up N = 113** | **Male N = 112** | **Female N = 108** |
| **Q1** | Your health information is divided into several different sections (e.g. diagnoses test results and images such as x-rays or scans). Would you like to: | Share all information | 35 (71) | 41 (95) | 36 (73) | 39 (83) | 3 (50) | 23 (92) | 17 (81) | 69 (79) | 91 (82) | 98 (89) | 79 (74) |
|  |  | Share no information | 6 (12) | 1 (2) | 3 (6) | 1 (2) | 1 (17) | 0 (0) | 2 (10) | 8 (9) | 2 (2) | 4 (4) | 7 (7) |
|  |  | Share specific information | 8 (16) | 1 (2) | 10 (20) | 7 (15) | 2 (33) | 2 (8) | 2 (10) | 10 (11) | 18 (16) | 8 (7) | 21 (20) |
|  |  | No response | 0 | 2 | 0 | 1 | 0 | 0 | 1 | 0 | 2 | 2 | 1 |
| **Q2** | Your biological samples are classified into several different types (e.g. blood urine tissues). Would you like to: | Share all information | 35 (71) | 40 (93) | 36 (73) | 37 (79) | 3 (50) | 22 (88) | 18 (86) | 68 (78) | 87 (78) | 95 (86) | 78 (73) |
|  |  | Share no information | 7 (14) | 2 (5) | 3 (6) | 4 (9) | 2 (33) | 0 (0) | 2 (10) | 8 (9) | 8 (7) | 6 (5) | 11 (10) |
|  |  | Share specific information | 7 (14) | 1 (2) | 10 (20) | 6 (13) | 1 (17) | 3 (12) | 1 (5) | 11 (13) | 16 (14) | 9 (8) | 18 (17) |
|  |  | No response | 0 | 2 | 0 | 1 | 0 | 0 | 1 | 0 | 2 | 2 | 1 |
| **Q3** | There are many different areas of medical research (e.g. research on cancer diabetes reproductive disorders genetic disorders heart disease etc.). Would you like to: | Share all information | 33 (67) | 40 (91) | 39 (81) | 35 (74) | 5 (83) | 20 (83) | 18 (86) | 67 (78) | 87 (78) | 96 (88) | 76 (71) |
|  |  | Share no information | 6 (12) | 1 (2) | 1 (2) | 0 (0) | 1 (17) | 0 (0) | 1 (5) | 7 (8) | 1 (1) | 3 (3) | 5 (5) |
|  |  | Share specific information | 10 (20) | 3 (7) | 8 (17) | 12 (26) | 0 (0) | 4 (17) | 2 (10) | 12 (14) | 23 (21) | 10 (9) | 26 (24) |
|  |  | No response | 0 | 1 | 1 | 1 | 0 | 1 | 1 | 1 | 2 | 3 | 1 |
| **Q4** | When asked for consent to share your information or samples would you like to have an option to think about the decision and be asked again later? | Yes | 24 (49) | 18 (42) | 25 (52) | 30 (63) | 0 (0) | 10 (42) | 9 (43) | 35 (41) | 63 (57) | 51 (47) | 54 (50) |
|  |  | No | 25 (51) | 25 (58) | 23 (48) | 18 (38) | 6 (100) | 14 (58) | 12 (57) | 51 (59) | 48 (43) | 57 (53) | 54 (50) |
|  |  | No response | 0 | 2 | 1 | 0 | 0 | 1 | 1 | 1 | 2 | 4 | 0 |
| **Q5** | Your health information and samples are often requested for future studies. Would you like to: | Broad consent | 26 (55) | 28 (67) | 25 (53) | 20 (43) | 4 (67) | 16 (67) | 11 (58) | 49 (60) | 59 (53) | 65 (61) | 54 (51) |
|  |  | Study-specific consent | 15 (32) | 13 (31) | 20 (43) | 26 (55) | 0 (0) | 6 (25) | 6 (32) | 24 (29) | 50 (45) | 37 (35) | 42 (40) |
|  |  | Would not share | 6 (13) | 1 (2) | 2 (4) | 1 (2) | 2 (33) | 2 (8) | 2 (11) | 9 (11) | 3 (3) | 4 (4) | 9 (9) |
|  |  | No response | 2 | 3 | 2 | 1 | 0 | 1 | 3 | 5 | 1 | 6 | 3 |
| **Q6** | A CONTACT POOL may be created with patient names phone numbers and key pieces of health information. UHN Researchers with ethical approval for their studies could search this pool to find participants. Would you prefer to be: | Asked for permission | 33 (67) | 24 (57) | 33 (70) | 34 (72) | 3 (50) | 7 (30) | 9 (50) | 48 (57) | 77 (69) | 65 (62) | 67 (63) |
|  |  | Automatically entered | 16 (33) | 18 (43) | 14 (30) | 13 (28) | 3 (50) | 16 (70) | 9 (50) | 36 (43) | 35 (31) | 40 (38) | 40 (37) |
|  |  | No response | 0 | 3 | 2 | 1 | 0 | 2 | 4 | 3 | 1 | 7 | 1 |
| **Q7** | How comfortable are you with providing consent for your information or samples to be shared with Researchers within UHN? | Very Comfortable | 27 (55) | 32 (71) | 23 (48) | 23 (48) | 3 (50) | 13 (52) | 11 (50) | 49 (56) | 61 (54) | 63 (56) | 58 (54) |
|  |  | Comfortable | 13 (27) | 8 (18) | 16 (33) | 12 (25) | 2 (33) | 11 (44) | 4 (18) | 23 (26) | 35 (31) | 32 (29) | 29 (27) |
|  |  | Neutral | 6 (12) | 4 (9) | 8 (17) | 10 (21) | 1 (17) | 1 (4) | 6 (27) | 10 (11) | 14 (13) | 14 (13) | 16 (15) |
|  |  | Uncomfortable | 1 (2) | 1 (2) | 1 (2) | 2 (4) | 0 (0) | 0 (0) | 1 (5) | 3 (3) | 1 (1) | 3 (3) | 2 (2) |
|  |  | Very Uncomfortable | 2 (4) | 0 (0) | 0 (0) | 1 (2) | 0 (0) | 0 (0) | 0 (0) | 2 (2) | 1 (1) | 0 (0) | 2 (2) |
|  |  | No response | 0 | 0 | 1 | 0 | 0 | 0 | 0 | 0 | 1 | 0 | 1 |
| **Q8** | How comfortable are you with providing consent for your information or samples to be shared with Researchers at other hospital-based research institutes? | Very Comfortable | 23 (47) | 26 (58) | 13 (27) | 12 (25) | 2 (33) | 9 (36) | 6 (27) | 36 (41) | 43 (38) | 44 (39) | 41 (38) |
|  |  | Comfortable | 11 (22) | 10 (22) | 15 (31) | 18 (38) | 1 (17) | 13 (52) | 4 (18) | 23 (26) | 41 (36) | 35 (31) | 33 (31) |
|  |  | Neutral | 10 (20) | 7 (16) | 12 (24) | 11 (23) | 2 (33) | 2 (8) | 9 (41) | 18 (21) | 17 (15) | 21 (19) | 23 (21) |
|  |  | Uncomfortable | 3 (6) | 2 (4) | 6 (12) | 5 (10) | 0 (0) | 1 (4) | 2 (9) | 6 (7) | 9 (8) | 8 (7) | 8 (7) |
|  |  | Very Uncomfortable | 2 (4) | 0 (0) | 3 (6) | 2 (4) | 1 (17) | 0 (0) | 1 (5) | 4 (5) | 3 (3) | 4 (4) | 3 (3) |
| **Q9** | How comfortable are you with providing consent for your information or samples to be shared with Researchers at universities? | Very Comfortable | 19 (39) | 21 (48) | 12 (24) | 12 (25) | 1 (17) | 6 (27) | 5 (23) | 29 (35) | 37 (33) | 37 (34) | 34 (31) |
|  |  | Comfortable | 15 (31) | 13 (30) | 14 (29) | 23 (48) | 2 (33) | 15 (68) | 7 (32) | 35 (42) | 40 (36) | 42 (39) | 40 (37) |
|  |  | Neutral | 9 (18) | 7 (16) | 12 (24) | 9 (19) | 2 (33) | 0 (0) | 5 (23) | 13 (15) | 21 (19) | 18 (17) | 20 (19) |
|  |  | Uncomfortable | 4 (8) | 2 (5) | 9 (18) | 2 (4) | 1 (17) | 0 (0) | 4 (18) | 4 (5) | 10 (9) | 8 (7) | 10 (9) |
|  |  | Very Uncomfortable | 2 (4) | 1 (2) | 2 (4) | 2 (4) | 0 (0) | 1 (5) | 1 (5) | 3 (4) | 4 (4) | 3 (3) | 4 (4) |
|  |  | No response | 0 | 1 | 0 | 0 | 0 | 3 | 0 | 3 | 1 | 4 | 0 |
| **Q10** | How comfortable are you with providing consent for your information or samples to be shared with For-profit businesses (e.g. drug or insurance companies such as Pfizer)? | Very Comfortable | 11 (22) | 15 (34) | 3 (6) | 3 (6) | 0 (0) | 3 (13) | 2 (9) | 18 (21) | 15 (13) | 20 (18) | 15 (14) |
|  |  | Comfortable | 6 (12) | 3 (7) | 5 (10) | 4 (8) | 1 (17) | 5 (22) | 0 (0) | 15 (18) | 9 (8) | 13 (12) | 11 (10) |
|  |  | Neutral | 9 (18) | 7 (16) | 11 (22) | 14 (29) | 1 (17) | 7 (30) | 6 (27) | 21 (25) | 22 (20) | 24 (22) | 25 (23) |
|  |  | Uncomfortable | 11 (22) | 10 (23) | 10 (20) | 12 (25) | 1 (17) | 7 (30) | 7 (32) | 15 (18) | 29 (26) | 26 (24) | 25 (23) |
|  |  | Very Uncomfortable | 12 (24) | 9 (20) | 20 (41) | 15 (31) | 3 (50) | 1 (4) | 7 (32) | 16 (19) | 37 (33) | 26 (24) | 32 (30) |
|  |  | No response | 0 | 1 | 0 | 0 | 0 | 2 | 0 | 2 | 1 | 3 | 0 |
| **Q11** | How comfortable are you with providing consent for your information or samples to be shared with Not-for-profit businesses (e.g. Heart and Stroke Foundation of Canada)? | Very Comfortable | 17 (35) | 20 (45) | 12 (26) | 10 (21) | 1 (17) | 4 (16) | 4 (18) | 26 (31) | 34 (30) | 37 (33) | 27 (25) |
|  |  | Comfortable | 8 (16) | 8 (18) | 17 (36) | 11 (23) | 2 (33) | 15 (60) | 4 (18) | 29 (34) | 28 (25) | 33 (30) | 28 (26) |
|  |  | Neutral | 13 (27) | 9 (20) | 10 (21) | 15 (31) | 1 (17) | 2 (8) | 9 (41) | 18 (21) | 23 (21) | 21 (19) | 28 (26) |
|  |  | Uncomfortable | 7 (14) | 3 (7) | 5 (11) | 11 (23) | 2 (33) | 3 (12) | 2 (9) | 9 (11) | 20 (18) | 14 (13) | 17 (16) |
|  |  | Very Uncomfortable | 4 (8) | 4 (9) | 3 (6) | 1 (2) | 0 (0) | 1 (4) | 3 (14) | 3 (4) | 7 (6) | 6 (5) | 6 (6) |
|  |  | No response | 0 | 1 | 2 | 0 | 0 | 0 | 0 | 2 | 1 | 1 | 2 |
| **Q12** | How comfortable are you with providing consent for your information or samples to be shared Provincially (i.e. within Ontario)? | Very Comfortable | 13 (27) | 18 (41) | 9 (19) | 6 (13) | 1 (17) | 5 (20) | 4 (18) | 24 (28) | 24 (21) | 32 (29) | 20 (19) |
|  |  | Comfortable | 12 (24) | 9 (20) | 15 (32) | 13 (27) | 1 (17) | 15 (60) | 5 (23) | 28 (33) | 32 (29) | 31 (28) | 34 (32) |
|  |  | Neutral | 12 (24) | 10 (23) | 12 (26) | 20 (42) | 2 (33) | 2 (8) | 6 (27) | 19 (22) | 33 (29) | 27 (24) | 31 (29) |
|  |  | Uncomfortable | 5 (10) | 3 (7) | 8 (17) | 8 (17) | 2 (33) | 2 (8) | 5 (23) | 8 (9) | 15 (13) | 15 (14) | 13 (12) |
|  |  | Very Uncomfortable | 7 (14) | 4 (9) | 3 (6) | 1 (2) | 0 (0) | 1 (4) | 2 (9) | 6 (7) | 8 (7) | 6 (5) | 8 (8) |
|  |  | No response | 0 | 1 | 2 | 0 | 0 | 0 | 0 | 2 | 1 | 1 | 2 |
| **Q13** | How comfortable are you with providing consent for your information or samples to be shared Nationally (i.e. within Canada)? | Very Comfortable | 13 (27) | 20 (45) | 7 (15) | 6 (13) | 1 (17) | 5 (20) | 3 (14) | 24 (28) | 25 (23) | 32 (29) | 20 (19) |
|  |  | Comfortable | 12 (24) | 10 (23) | 13 (28) | 14 (29) | 1 (17) | 14 (56) | 5 (23) | 26 (30) | 33 (30) | 30 (27) | 34 (32) |
|  |  | Neutral | 11 (22) | 9 (20) | 13 (28) | 19 (40) | 2 (33) | 3 (12) | 7 (32) | 19 (22) | 31 (28) | 26 (24) | 31 (29) |
|  |  | Uncomfortable | 6 (12) | 4 (9) | 10 (21) | 6 (13) | 1 (17) | 2 (8) | 5 (23) | 10 (12) | 14 (13) | 16 (15) | 12 (11) |
|  |  | Very Uncomfortable | 7 (14) | 1 (2) | 4 (9) | 3 (6) | 1 (17) | 1 (4) | 2 (9) | 7 (8) | 8 (7) | 6 (5) | 10 (9) |
|  |  | No response | 0 | 1 | 2 | 0 | 0 | 0 | 0 | 1 | 2 | 2 | 1 |
| **Q14** | How comfortable are you with providing consent for your information or samples to be shared Internationally (i.e. around the world)? | Very Comfortable | 12 (24) | 17 (39) | 6 (13) | 5 (10) | 0 (0) | 4 (16) | 3 (14) | 21 (24) | 20 (18) | 25 (23) | 19 (18) |
|  |  | Comfortable | 6 (12) | 6 (14) | 7 (15) | 10 (21) | 1 (17) | 11 (44) | 1 (5) | 21 (24) | 19 (17) | 24 (22) | 17 (16) |
|  |  | Neutral | 12 (24) | 10 (23) | 10 (21) | 14 (29) | 3 (50) | 4 (16) | 6 (27) | 16 (19) | 31 (28) | 25 (23) | 28 (26) |
|  |  | Uncomfortable | 9 (18) | 6 (14) | 13 (28) | 13 (27) | 1 (17) | 4 (16) | 8 (36) | 16 (19) | 22 (20) | 20 (18) | 26 (24) |
|  |  | Very Uncomfortable | 10 (20) | 5 (11) | 11 (23) | 6 (13) | 1 (17) | 2 (8) | 4 (18) | 12 (14) | 19 (17) | 16 (15) | 17 (16) |
|  |  | No response | 0 | 1 | 2 | 0 | 0 | 0 | 0 | 1 | 2 | 2 | 1 |
| **Q15** | Sometimes for-profit companies develop partnerships with UHN and we work together on medical research projects. How comfortable are you consenting to share your information or samples for these projects? | Very Comfortable | 10 (20) | 16 (36) | 4 (8) | 5 (10) | 0 (0) | 3 (12) | 3 (14) | 17 (20) | 18 (16) | 23 (21) | 15 (14) |
|  |  | Comfortable | 12 (24) | 11 (24) | 18 (37) | 14 (29) | 2 (33) | 16 (64) | 6 (27) | 36 (41) | 31 (27) | 37 (33) | 36 (33) |
|  |  | Neutral | 10 (20) | 8 (18) | 11 (22) | 13 (27) | 3 (50) | 3 (12) | 7 (32) | 16 (18) | 25 (22) | 23 (21) | 25 (23) |
|  |  | Uncomfortable | 9 (18) | 3 (7) | 8 (16) | 10 (21) | 0 (0) | 3 (12) | 4 (18) | 7 (8) | 22 (19) | 16 (14) | 16 (15) |
|  |  | Very Uncomfortable | 8 (16) | 7 (16) | 8 (16) | 6 (13) | 1 (17) | 0 (0) | 2 (9) | 11 (13) | 17 (15) | 13 (12) | 16 (15) |
| **Q16** | Sometimes for-profit companies ask UHN for health information or samples. How comfortable are you consenting to share your information or samples with these companies if UHN is not directly involved in their work? | Very Comfortable | 4 (9) | 13 (30) | 3 (6) | 2 (4) | 0 (0) | 3 (12) | 2 (9) | 12 (14) | 11 (10) | 18 (16) | 7 (7) |
|  |  | Comfortable | 9 (20) | 10 (23) | 6 (13) | 8 (17) | 1 (17) | 9 (36) | 3 (14) | 24 (29) | 16 (15) | 27 (24) | 16 (16) |
|  |  | Neutral | 7 (16) | 7 (16) | 9 (19) | 11 (23) | 1 (17) | 2 (8) | 6 (27) | 14 (17) | 17 (16) | 17 (15) | 20 (20) |
|  |  | Uncomfortable | 11 (24) | 4 (9) | 12 (26) | 14 (29) | 2 (33) | 10 (40) | 7 (32) | 16 (19) | 30 (28) | 23 (21) | 30 (29) |
|  |  | Very Uncomfortable | 14 (31) | 10 (23) | 17 (36) | 13 (27) | 2 (33) | 1 (4) | 4 (18) | 18 (21) | 35 (32) | 26 (23) | 29 (28) |
|  |  | No response | 4 | 1 | 2 | 0 | 0 | 0 | 0 | 3 | 4 | 1 | 6 |
| **Q17** | Sometimes medical research using health information or samples at UHN leads to discoveries that are commercialized and sold for-profit in the future. How do you feel about consenting to share your information or samples being involved in this? | Very Comfortable | 8 (16) | 13 (29) | 2 (4) | 2 (4) | 0 (0) | 4 (17) | 2 (10) | 14 (16) | 13 (12) | 19 (17) | 10 (9) |
|  |  | Comfortable | 10 (20) | 8 (18) | 14 (29) | 9 (19) | 1 (17) | 10 (42) | 3 (14) | 28 (33) | 21 (19) | 31 (28) | 21 (20) |
|  |  | Neutral | 12 (24) | 11 (24) | 11 (22) | 12 (26) | 2 (33) | 3 (13) | 7 (33) | 19 (22) | 25 (22) | 26 (23) | 25 (23) |
|  |  | Uncomfortable | 8 (16) | 6 (13) | 10 (20) | 15 (32) | 2 (33) | 6 (25) | 5 (24) | 12 (14) | 30 (27) | 20 (18) | 27 (25) |
|  |  | Very Uncomfortable | 11 (22) | 7 (16) | 12 (24) | 9 (19) | 1 (17) | 1 (4) | 4 (19) | 13 (15) | 24 (21) | 15 (14) | 24 (22) |
|  |  | No response | 0 | 0 | 0 | 1 | 0 | 1 | 1 | 1 | 0 | 1 | 1 |
| **Q18** | Would you like to be able to track who is using your information or samples and what they are using it for? | Yes | 29 (60) | 24 (53) | 33 (67) | 35 (73) | 3 (50) | 10 (40) | 11 (50) | 42 (48) | 81 (72) | 62 (55) | 71 (66) |
|  |  | No | 14 (29) | 20 (44) | 16 (33) | 11 (23) | 2 (33) | 14 (56) | 9 (41) | 39 (45) | 29 (26) | 47 (42) | 30 (28) |
|  |  | Not applicable. I would not share at all. | 5 (10) | 1 (2) | 0 (0) | 2 (4) | 1 (17) | 1 (4) | 2 (9) | 6 (7) | 2 (2) | 3 (3) | 6 (6) |
|  |  | No response | 1 | 0 | 0 | 0 | 0 | 0 | 0 | 0 | 1 | 0 | 1 |
| **Q19** | Would you like to be notified with the results of studies that have used your information or samples? | Yes | 35 (71) | 31 (69) | 35 (71) | 35 (73) | 4 (67) | 15 (60) | 12 (55) | 52 (60) | 91 (81) | 75 (67) | 79 (73) |
|  |  | No | 9 (18) | 13 (29) | 14 (29) | 11 (23) | 1 (17) | 9 (36) | 8 (36) | 29 (33) | 20 (18) | 34 (30) | 23 (21) |
|  |  | Not applicable. I would not share at all. | 5 (10) | 1 (2) | 0 (0) | 2 (4) | 1 (17) | 1 (4) | 2 (9) | 6 (7) | 2 (2) | 3 (3) | 6 (6) |
| **Q20** | If you do want to be notified of study results how would you like to be notified? | Online via an electronic patient portal | 22 (45) | 11 (26) | 13 (27) | 18 (38) | 2 (33) | 10 (40) | 6 (27) | 26 (30) | 44 (39) | 35 (32) | 40 (37) |
|  |  | Online via email | 10 (20) | 16 (37) | 8 (16) | 13 (27) | 0 (0) | 3 (12) | 0 (0) | 19 (22) | 31 (28) | 24 (22) | 26 (24) |
|  |  | Standard mail | 9 (18) | 6 (14) | 14 (29) | 8 (17) | 2 (33) | 4 (16) | 6 (27) | 15 (17) | 22 (20) | 21 (19) | 22 (20) |
|  |  | I do NOT want to be notified of study results | 3 (6) | 9 (21) | 12 (24) | 9 (19) | 1 (17) | 6 (24) | 8 (36) | 18 (21) | 14 (13) | 26 (24) | 14 (13) |
|  |  | Not applicable. I would not share at all | 5 (10) | 1 (2) | 2 (4) | 0 (0) | 1 (17) | 2 (8) | 2 (9) | 8 (9) | 1 (1) | 4 (4) | 6 (6) |
|  |  | No response | 0 | 2 | 0 | 0 | 0 | 0 | 0 | 1 | 1 | 2 | 0 |
